# A participatory approach for building *ex ante* impact pathways towards a prudent use of antimicrobials in pig and poultry sectors in France

**DOI:** 10.1371/journal.pone.0277487

**Published:** 2022-11-15

**Authors:** Marie-Jeanne Guenin, Catherine Belloc, Christian Ducrot, Aurelle de Romémont, Marisa Peyre, Sophie Molia

**Affiliations:** 1 UMR ASTRE, Université de Montpellier, CIRAD, INRAE, Montpellier, France; 2 INRAE, Oniris, BIOEPAR, Nantes, France; 3 CIRAD-DIMS, Montpellier, France; 4 UMR Innovation, Montpellier, France; University of Liverpool & International Livestock Research Institute (ILRI), UNITED KINGDOM

## Abstract

Antimicrobial resistance (AMR) is a global public health threat responsible for 700,000 deaths per year worldwide. There is scientific evidence of the causal relationship between antimicrobial use (AMU) along the food chain and AMR. Improving AMU in livestock is therefore a key component in the fight against AMR. To improve AMU in livestock, there is no one-size-fits-all solution and strategies must be context-adapted and socially acceptable for actors in order to increase AMU sustainability. AMU decision-making is based on an interdependent set of economic, behavioral, ethical, and cultural factors that need to be assessed to advise on the potential impacts of measures. We hypothesized that a participatory strategic planning approach may increase the plausibility and the efficacy of the strategies formulated by facilitating the dialogue between actors of diverse backgrounds, stimulating innovative thinking and constant considerations of contextual factors, actors and impacts. We adapted and applied the ImpresS *ex ante* approach (IMPact in reSearch in the South, https://impress-impact-recherche.cirad.fr/) within a Living Lab engaging actors from the French pig and poultry sectors in co-creation of innovative strategies towards improved AMU. We conducted semi-structured interviews and participatory workshops between April 2021 and March 2022. The results describe 1) an initial diagnosis of the current AMU situation in the pig and poultry sectors in France; 2) a common vision of the future to which participants would like to contribute through the intervention; 3) an identification of the current problems opposed to this vision of the future; 4) a defined scope of the intervention; 5) a typology of actors protagonist or impacted by those issues and 6) outcome maps to solve a priority problem related to indicators and monitoring. This study provides recommendations for decision-makers on plausible and innovative strategies to sustainably improve AMU in pig and poultry sectors in France and evidence of the benefits of participatory strategic planning approaches.

## 1. Introduction

Antimicrobial resistance (AMR) is a global public health threat responsible for 700,000 deaths per year worldwide, a toll that has the potential to rise to 10 million by 2050 [1]. Even if the quantification of the burden of AMR in public health attributable to antimicrobial use (AMU) on farms remains challenging, there is ample scientific evidence of this causal relationship and of the human population exposure to antimicrobial-resistant pathogens via the food chain or the environment [2–4]. The last century has been marked by the misuse and overuse of antimicrobials (AMs) in animal production, such as AMU for disease prevention and as growth promoters, contributing to AMR emergence and spread while allowing industrialization of farming [5, 6]. The intensification of production systems to meet the growing consumer demand for animal protein could increase by 67% the global AMU in livestock between 2010 and 2030 and sales of AMs are expected to rise by 11.5% from 2017 to 2030 [7, 8]. The lack of investment in the discovery and development of new AM agents and alternatives increase the AMR threat [9, 10]. Improving AMU in animal production and integrating a One Health approach is therefore a key component in the fight against AMR [11].

To tackle the AMR issue, many initiatives and strategies have been implemented to reduce AMU in animal production but they were not all entirely successful [12]. Barriers and levers to implement and adopt AMR risk mitigation policies differ in different agricultural settings around the world [13]. To improve AMU in livestock while ensuring animal health and welfare, there is no one-size-fits-all solution and strategies must be context-adapted and socially acceptable for the animal health and production actors and the general public in order to increase their chances of success. Therefore, customized and optimized approaches including a varying mixture of strategies need to be explored while considering their potential risks and benefits in a particular setting and in full consideration of a range of actors’ values [13–15].

Changes in AMU practices occur in specific settings but are also nested in broader logics and a global sociotechnical system. Trigger events such as health problems, changes in professional networks, shifts of economic and technical objectives, as well as medium- to long-term processes such as modifications of actors’ experiences and practices, can influence the transition pathways towards reduced AMU. In this context, multi- and transdisciplinary research involving social sciences and concerted approaches between different actors are needed to understand and promote the variability of the dynamics of AMU reduction, the interactions between actors and the collective actions implemented to tackle the challenge of reducing AMU [14–16].

There is a major need to try different forms of large- and small-scale interventions and to document their negative and positive effects. Many studies assess the long-term effects after intervention implementation to estimate its success [17–23]. AMU decision-making is based on an interdependent set of economic, behavioral, ethical, and cultural factors that need to be assessed to advise policy-makers on the potential impact of regulations [24]. To our knowledge, the impacts generated by interventions in animal health are only assessed through *ex post* evaluation but are not considered *ex ante* as a starting point to identify potential strategies and to build action plans.

Impact evaluation has been increasingly used over the past 30 years to evaluate interventions in the fields of development, policy-making, and research. It involves assessing the positive and negative, intended and unintended, direct and indirect, primary and secondary long-term effects on ultimate beneficiaries that result from an intervention but also, in some cases, assessing who were impacted, how, and why [25]. In agriculture-related interventions, the challenge of impact evaluation is to identify impacts that go beyond economic impacts (e.g. social, territorial, environmental, political, and health-related impacts) taking into account the complexity of interventions and underlying causality links. This has become more difficult as agricultural research and innovation systems are increasingly open, complex, and rapidly changing [26]. Participatory approaches to impact evaluation tend to involve different stakeholders, including beneficiaries, at all stages of the intervention. Stakeholders are involved in both identifying the changes they wish to see, and assessing whether, and how, those changes have been reached and what are the contribution of the intervention they are connected to [25, 27].

We hypothesized that the use of a participatory strategic planning approach that considers contextual factors, actors and impacts since the beginning of the intervention design is likely to increase the plausibility of the assumptions and the efficacy of the strategies formulated. We adapted and applied the ImpresS *ex ante* (https://impress-impact-recherche.cirad.fr/) approach within a Living Lab (LL) engaging French actors from the pig and poultry sectors in co-creating, validating, testing in real life contexts and evaluating innovative strategies towards improved AMU. We assumed that this *ex ante* participatory impact pathway building approach may be complementary with the LL process by facilitating the dialogue between actors of diverse backgrounds, stimulating innovative and evaluative thinking and constant considerations of contextual factors and impacts of the innovation [28]. We describe the method used, the results obtained and the lessons learned.

## 2. Method

### 2.1 The ImpresS *ex ante* approach: A participatory process to build a shared vision of change

**Fig 1:**
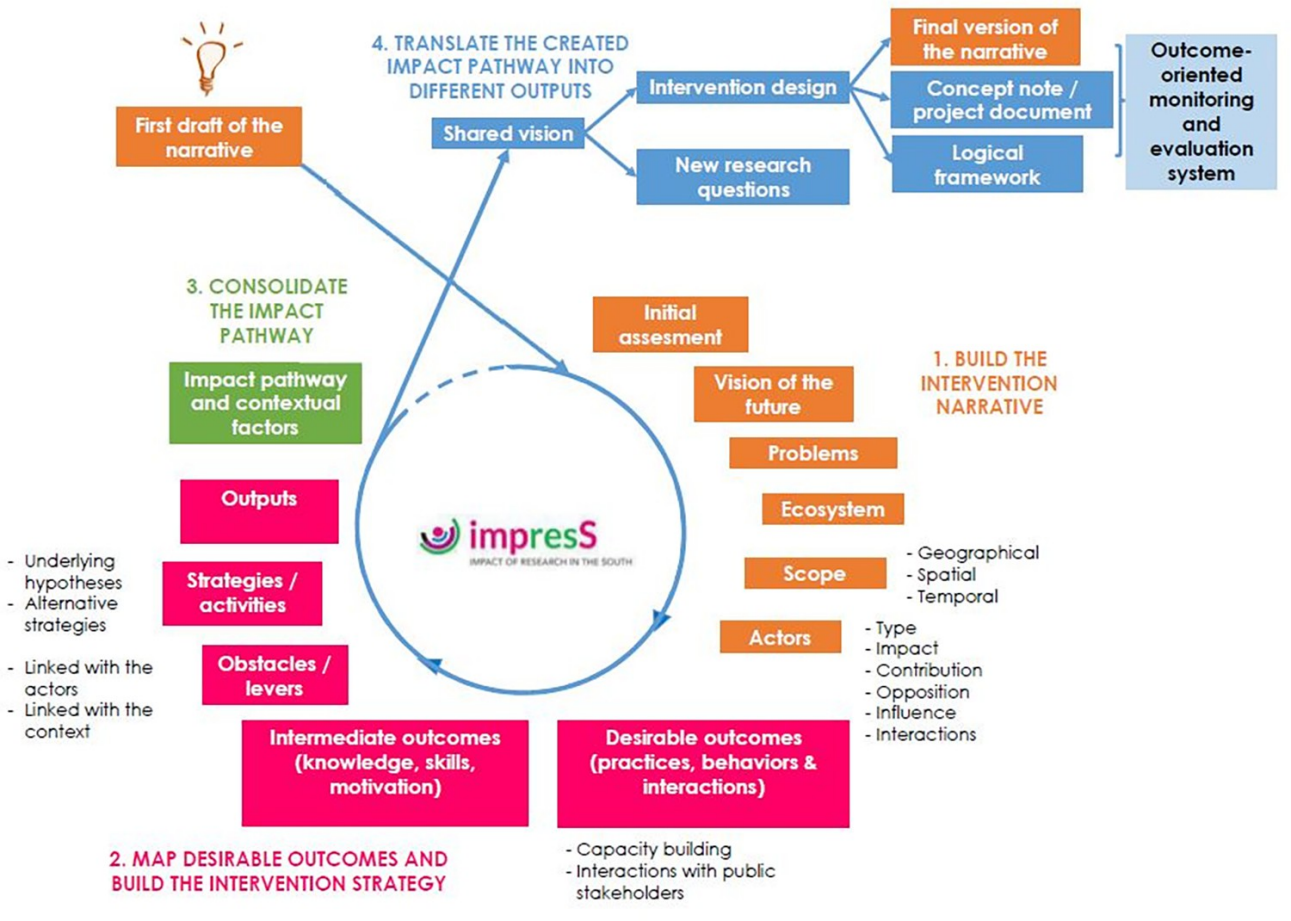
The four stages of the ImpresS *ex ante* approach. Figure extracted from the ImpresS *ex ante* methodological guide to *ex ante* co-construction of development-oriented research impact pathways (second version), https://doi.org/10.19182/agritrop/00147. This document is provided under the terms of the Creative Commons License CC-BY 4.0: Attribution 4.0 International https://creativecommons.org/licenses/by/4.0/deed.fr.

The ImpresS *ex ante* approach is a participatory *ex ante* impact pathway building approach, developed by the French Agricultural Research Center for International Development (CIRAD) and inspired by existing theorical frameworks such as outcome mapping, applications of theory of change building and program theory respectively described by Earl et al., Mayne et al., Alvarez et al., and Funnell and Rogers [29–33]. This approach aims to elucidate a collective and shared vision of the logic of an intervention, that includes all the strategies and actions structured around a common intention, through the construction of an impact pathway underpinned by a theory of change [31, 34, 35]. The impact pathway describes the underlying program theory of the intervention by elucidating the causal links between resources mobilized by the intervention (inputs), the intervention’s products (outputs), the changes in practices, behavior and interactions of the actors associated with the use, adaptation or transformation of these outputs (desirable changes or outcomes) and the impacts to which these outcomes contribute in the long term. To increase the success of an intervention in terms of desirable changes and long-term impacts achievement, this approach considers the contextual factors including actors, the ecosystem of interventions and the innovation trajectory related to the issue. This approach is usually deployed to design and manage research for development projects or research networks. Nevertheless, the method may be consistent and complementary with other participatory innovation approaches such as LLs, that is an open-innovation research approach aimed at involving end-users or their representatives in the co-creation, exploration, experimentation and evaluation of innovative scenarios and technologies in their real-life context and considering their potential usefulness, adoption and impacts [28]. The impact pathway is a conceptual model of a shared vision of the intervention logic, used mainly for strategic planning, that can be later translated into a collective action plan for the experimentation phase and into an intervention monitoring and evaluation system based on relevant outcomes and impacts indicators for the evaluation phase in a LL. The ImpresS *ex ante* approach is an iterative and adaptative process including the four following stages: 1) Building a shared vision of the intervention narrative; 2) Mapping the desirable outcomes and building the intervention strategy; 3) Consolidating the intervention impact pathway; 4) Translating the impact pathway into different outputs to fulfill the objective of the project, reflecting the project logic and facilitating its collective implementation. The approach is highly participative in order to enable different actors to exchange, debate and decide to which changes they want to contribute collectively and how. It is also flexible and adaptative to users’ objectives, resources and timeframe. We deployed and adapted the two first stages following the different steps described in the ImpresS *ex ante* Methodological Guide (Fig 1) [29].

### 2.2 Selection of the participants for the participatory process

Reducing AMU requires combined actions at different scales [16]. In highly organized sectors, such as in pig and poultry industry, the challenge is to organize this coherence of actions and efforts made by all the actors of the supply chain. The scale of production organizations and sectors is therefore relevant to consider the issues related to the performance of the industry, market dynamics and bargaining power of each profession they represent. It is also relevant to consider the dynamics related to the veterinary profession and to involve the State which strongly contributed to put in synergy in the various initiatives and to bring financial support [15]. From January to April 2021, to increase the plausibility of the intervention impact pathway, we therefore identified and involved representatives from the pig and poultry setors, the veterinary profession and Ministry of Agriculture and Food in the participatory process [36]. Each participant represents a national organization that may have different knowledge and viewpoints concerning the AMU situation in the French pig and poultry sectors and that can play a role in the innovation process towards improved AMU. We assumed that these similarly organized sectors faced the same AMU-related issues and therefore we decided to both include them in this LL and to separate them during the participatory process if this assumption was not relevant. The initial group of participants included two representatives of the pig (Institut du Porc, IFIP) and poultry (Institut Technique de l’Aviculture, ITAVI) technical institutes which carry out research and development activities to support the sectors, two representatives of the pig (Interprofession Nationale Porcine, INAPORC) and poultry (Association Nationale Interprofessionnelle de la Volaille de chair, ANVOL) inter-branch organizations which represent all the links of the supply chain and defend their interests, two representatives of the pig and poultry commissions of the National Society of Veterinary Technical Groups (Société Nationale des Groupements Techniques Vétérinaires, SNGTV) and one representative of the National Union of Veterinary Consultants (Syndicat National des Vétérinaires Conseil, SNVECO) which represent the veterinary practitioners and advisors working in livestock and defend their interests. We also included one representative of the College of Veterinary Surgeons (Ordre National des Vétérinaires, ONV) and one representative of the General Directorate of Food of the Ministry of Agriculture and Food (Direction Générale de l’Alimentation, DGAL) which were respectively developing the Calypso project which aims to collect AM sales data on a digital platform and the third national Ecoantibio plan. We anticipated potential necessary changes related to market competition and consumption and wanted to include one representative of a consumers’ association to collect their perception on these points. Because of time and availability constraints, it was not possible to have this actor in the group. To respect the principles of openness and representativeness we proposed to include new actors during the process, with the consent of participants and if they identified the need for an additional expertise during the participatory process.

### 2.3 Description of the participatory process

**Fig 2:**
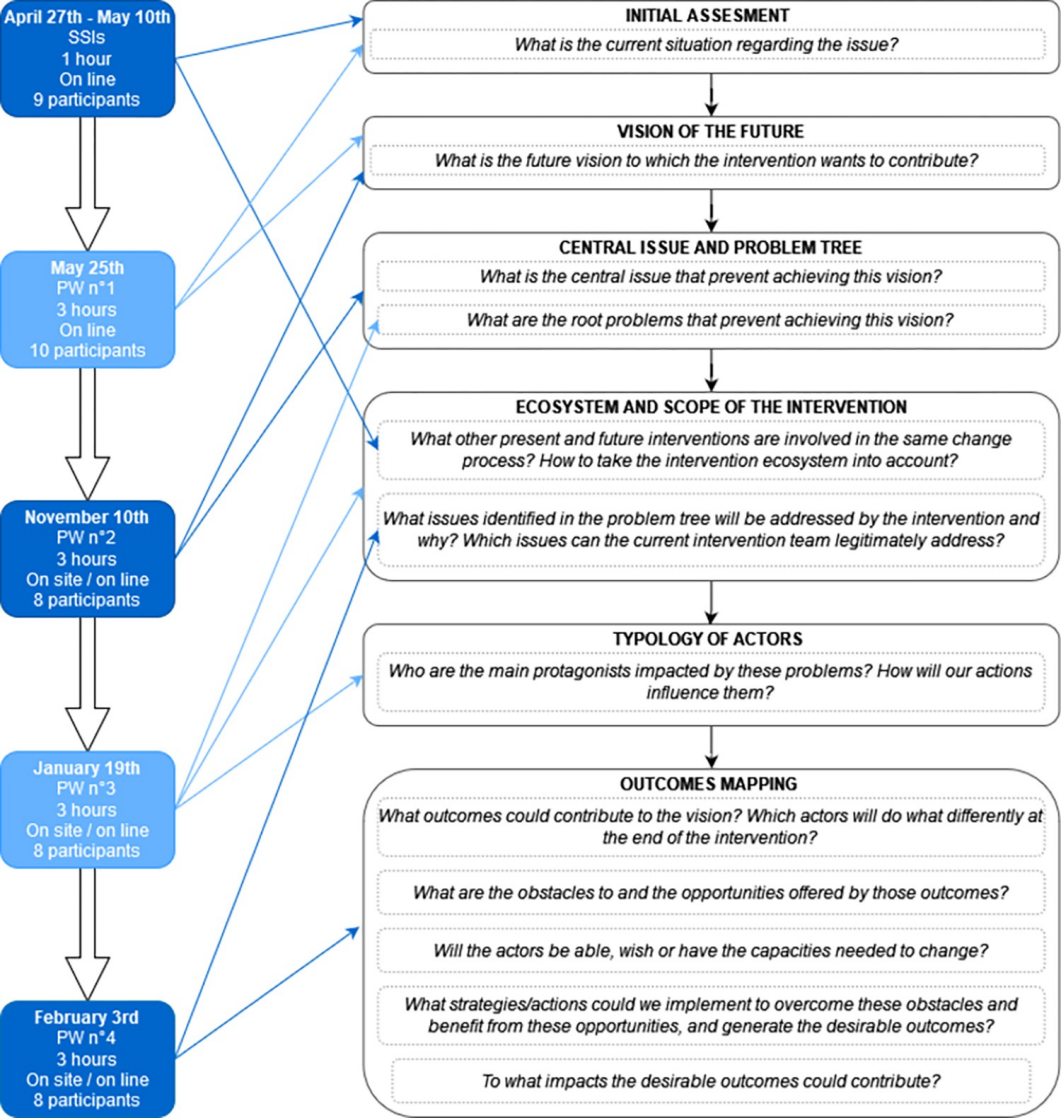
Timeline and modality of participatory activities to implement the ImpresS *ex ante* steps from April 2021 to March 2022. SSIs, semi-structured interviews; PW, participatory workshop. Inspired from the figure “A series of questions for formulating an intervention based on the vision of the future” in the ImpresS *ex ante* methodological guide to *ex ante* co-construction of development-oriented research impact pathways (second version), https://doi.org/10.19182/agritrop/00147.

The participatory process and related data collection were undertaken between April 2021 and March 2022 by a research team including one note-taker, one facilitator trained in participatory and ImpresS *ex ante* approaches and another facilitator with an expertise on the AMU situation in the pig and poultry sectors in France. Nine participants were solicited through individual semi-structured interviews (SSIs) and four participatory workshops (PWs) (Fig 2). The composition and the number of participants remained broadly stable throughout the participatory process. Because of agenda conflict, the representative of the pig inter-branch organization did not participate in the last three PWs and therefore only validated the initial assessment and contributed to the first version of the vision of the future. The representative of the poultry inter-branch organization did not participate to the first PW and subsequently gave his viewpoint on the initial assessment and the vision of the future during an individual interview and the second PW. The DGAL restructured its services at the beginning of the participatory process and the ownership of AMR-related projects changed. For the first PW, we included the heads of both the previous and the new office in charge of the AMR-related projects, to ease transition within the LL. Then, the project manager of AMR-related initiatives participated in the last three PWs.

#### 2.3.1 Initial assessment

Based on data from literature and individual SSIs, the research team, that included four professionals from the veterinary health field, produced an initial assessment of the current situation regarding AMU in France and particularly in pig and poultry sectors. We collected information on past and existing AMU reduction initiatives in terms of generated outcomes and impacts, and success or failure factors. This initial assessment was presented during the first PW and the participants enriched and validated a final version through a collective discussion.

#### 2.3.2 Vision of the future

The objective of this step was to collectively formulate a ten-year vision of the future to which the group of actors wished to contribute through the intervention they were co-constructing. During the first PW, each participant individually formulated two impacts that they wished to contribute to in the longer term; with the overall objective of “improving AMU in the pig and poultry sectors in France”. The individually identified impacts were collectively discussed with all the participants, who could agree or disagree or complete these ideas. The research team identified the points of convergence between the participants’ viewpoints and proposed a formulation of the ten-year vision of the future which was clarified and validated during the second PW by the participants.

#### 2.3.3 Central issue and problem tree

The objective of this step was to formulate the central issue, that is the main reason why the vision of the future is not yet reached. Based on the first PW the research team identified current problems mentioned by the participants and formulated a central issue which was clarified and validated during the second PW by the participants. We used the “problem tree” tool during the second and third PWs to identify the so-called problems, that are the underlying causes to this central issue, and their causal links [29].

#### 2.3.4 Ecosystem and scope of the intervention

During the third PW, the participants selected the problems they wished to address through the intervention. This selection was oriented by the legitimacy and confidence the participants felt towards being able to contribute to solving the identified problems. During the SSIs and PWs, we mapped the other existing and future interventions dealing with similar issues to consider potential synergies of work or to exclude some problems already addressed by other interventions. The research team proposed to group and reformulate interrelated and similar problems to avoid duplication. Based on the discussions and because of time constraints, we proposed a prioritization of the problems to work on during the next PW. The participants revised and validated these proposals during the fourth PW.

#### 2.3.5 Typology of actors

During the third PW, we asked participants to identify the actors who are protagonist and/or impacted by the problems included in the scope of the intervention and to characterize them as major, influential or positively or negatively impacted in relation to the issue identified by the group. Indeed, the originality of ImpresS *ex ante* is its actor-centered approach. In order to resolve problems in the longer term, it is not so much solutions that are considered but rather changes in actors’ practices, behavior and interactions. We asked the participants if the actors within the same category could be considered homogeneous in terms of interests, strategies and roles and to distinguish those who could be opposed or contribute to the intervention, to resolve the problems included in the scope of the intervention. This reflection on the actors allowed to better prepare the next step of the strategic planning process by reflecting on the roles they could play, the potential obstacles they might encounter or represent, and the way they might be impacted [29].

#### 2.3.6 Outcomes mapping

During the fourth PW, the participants started to identify the potential strategies and contributions to resolve the prioritized problems. We used the "outcomes mapping" tool to consecutively identify: 1) the desirable changes in terms of interactions, behaviors and practices for some actors to contribute to solve these problems (who should do what differently to resolve the targeted problems?); 2) the intermediate changes in terms of knowledge, capacities and motivations necessary to generate the above-mentioned changes in practices, behavior and interactions (can the targeted actors change, do they want to, do they know how?); 3) the current obstacles related to contextual factors or related to the actors themselves to generate these intermediate and final changes; 4) the strategies to overcome these obstacles; 5) the intervention outputs and activities to implement these strategies; 6) the negative and positive potential impacts to which the desirable outcomes might contribute to in the long term [29].

### 2.4 Data collection and analysis

All the SSIs and PWs were recorded and transcribed on Microsoft Word. We performed a thematic content analysis to extract the qualitative data from the transcripts and sort them in different broad themes on the NVivo qualitative data coding software [37]. Themes emerged from the reading and were different according to the objectives of the participatory activity and the step of the ImpresS *ex ante* approach. Workshop results such as impacts mapping, problem tree, actors mapping and outcomes mapping were documented using photographs or screenshots and reproduced on the Ayoa mind map software. To ensure the reliability of the data and of the construction process, we applied the iteration principle of the ImpresS *ex ante* approach. After each PW we analyzed data to produce reports sent to the participants for diffusion in their respective institutions and approval. Each meeting started by the presentation of the previous results and participants had the opportunity to collectively revise and validate them.

### 2.5 Ethics statement

Our study complies with the ethics requirements imposed by the European Commission to the ROADMAP project ©Grant Agreement Number 817626®. Ethical and scientific considerations have been controlled and validated by the funding European Research Executive Agency and the application of GDPR has been controlled by a Data Protection Officer. We provided information on the participation in the ROADMAP research project and the participants gave their oral and written consent. Respondents participated freely and anonymously to the research study.

## 3. Results

### 3.1 Initial assessment

The participants collectively assessed that the different previous interventions aiming to change AMU practices, such as the first and the second Ecoantibio plans and the regulation of the use of critically important AMs for treatment of human diseases, clearly contributed to a significant AMU reduction in the pig and poultry sectors in France [38]. The group agreed that the AMU decreasing trend reached a threshold of stagnation and questioned the opportunity to further decrease the amount of AMs used without negatively impacting animal health and welfare. The participants considered that AMU efforts should now target a “better” use rather than a “decreased” use. The participants assessed that the past interventions encouraged the emergence of various indicators which are necessary to monitor AMU evolution. However, these indicators and their calculation are not standardized [39]. These different interventions also influenced the emergence of different "antibiotic-free" labels with their own AMU monitoring systems and, in some cases, set of criteria related to animal welfare. The participants agreed that these charters contributed to AMU reductions but that they now represent an economic constraint for farmers when a curative AM treatment is necessary to ensure animal health and welfare. In light of these contextual factors, the challenge identified by the participants is to encourage a better AMU through appropriate and accepted actions while ensuring animal health and welfare and the economic viability of actors’ activities. A more precise AMU monitoring that would consider the diversity and the specific health situation of farms is perceived as an opportunity for improving AMU. The group also mentioned the need for incentive evidence of the positive impact of a good or reduced AMU on AMR.

This initial assessment encompassed the intervention in a broader ecosystem of projects and trajectories of change. In the European Union, the evolution of regulations according to AMU monitoring requires the reporting of sales and usage data. The participants identified possible synergies with the construction of the third Ecoantibio plan, which is concomitant with the revision of the One Health interministerial roadmap, and with the Calypso project led by the ONV that plans to collect data on AM sales at the farm level.

### 3.2 Vision of the future

The initial assessment provided a first insight of the impacts the group wished to contribute to within the next ten years through the intervention. The ten preliminary ideas on desirable impacts that the members of the group individually produced were highly convergent among participants. The collective discussion allowed to identify links between impacts and the first hypothetical strategies and outputs to generate them.

First, the participants collectively mentioned that the intervention contributes to the acceptability of practices oriented towards good AMU while ensuring animal health and welfare. They thought that this would require a continuous awareness-raising of AM users and end-users of animal products so that these actors understand the impact of AM misuse on animal health and welfare and on public health, and their influence through their treatment choices, herd management and consumption. This would also require to previously define what is good AMU respectful of animal health and welfare, to objectify through scientific data the link between AMU and AMR and the impacts on animal and human health, to enhance the One Health efforts already made and to develop appropriate communication tools to disseminate this information.

Secondly, the group mentioned that farmers, veterinarians and technicians, be involved together in the application of a more refined and adaptive AMU and in a more global approach of animal health and welfare. To this end, they would have and use indicators on AMU, other treatments, AMR, animal health and zootechnical parameters. These indicators and the way in which they are expressed should be standardized and listed in a specification to allow the farmer’s self-evaluation and the comparison on the basis of collective references. These indicators should be associated with consensual thresholds and applicable in the field. The data need to be recorded on digital tools. Laboratory tests, such as antibiogram, would provide data on AMR to adapt treatments. Farmers would need to have equipment, such as metering pumps, for proper AM administration. All actors would receive the same coherent message and be continuously trained in the use of these tools. In addition, farmers would be motivated by the technical and economic benefits to implement actions that improve the sanitary conditions (biosecurity measures, water quality, vaccination) on their farms.

Thirdly, the group mentioned that the veterinary and health network will be sustainable in the country and will provide the needed technical skills for better AMU and for health monitoring and management thanks to adapted solutions, that can be mandatory and at the initiative of the State, and thanks to the awareness of the economic value of the veterinary advisory service. Alternative medicines to AMs would be prescribed and delivered by veterinarians and their use would be regulated to control the potential risk of residues and cross-resistance.

Finally, the group wished that the better AMU approaches in the pig and poultry sectors in France be widespread and resist foreign competition thanks to their economic valorization. These approaches would be encouraged by the implementation of a precise framework and reference system on proper AMU that would allow derogations to the *stricto sensu* "antibiotic-free" label specifications. Consumers would influence these approaches by exercising their purchasing power and making informed consumption choices. To ensure better understanding by the consumer-citizen, this would require awareness-raising and standardization and clarification of labels.

Based on this discussion, we formulated a first version of the vision of the future that includes all the identified impacts and allowance to explore other hypothetical impact pathways. This formulation was clarified and validated during the second PW by the participants. The final version of the vision of the future is presented in Box 1.

**Box 1. The ten-year vision of the future regarding antimicrobial use in the pig and poultry sectors in France.**

“In 2031, in France, the proper use of antimicrobials in the poultry and pig sectors is a practice that focuses on "better" and not just "less", applied in all farms and accepted by actors involved in the use of antimicrobials (veterinarians, farmers, production organizations, pharmaceutical industries, purchasing centers, etc.) and by those involved in the use of animal products (slaughterhouses, distribution, restaurants, consumers, etc.). This practice, monitored by appropriate indicators, makes it possible to preserve the therapeutic arsenal while guaranteeing animal health and welfare on the one hand, and the sustainability of these sectors and of the veterinary network in the country on the other hand.”

### 3.3 Central issue and problem tree

Based on the first collective discussion, we had a preliminary idea of the barriers or of what is currently missing to generate the desired impacts. We formulated a first version of the central issue that was clarified and validated during the second PW by the participants. The final version of the central issue is presented in Box 2. During the second and third PWs the participants systematically identified forty-eight underlying causes to this central issue distributed in four thematic roots of problems related to consumption (section 3.3.1), competitiveness (section 3.3.2), indicators and monitoring (section 3.3.3), and barriers for veterinarians and farmers to change in practices (section 3.3.4).

**Box 2. The central issue regarding antimicrobial use in the pig and poultry sectors in France.**

“Pig and poultry consumption choices do not systematically take into account the use of antimicrobials by actors (veterinarians, farmers, production organizations, etc.) who lack or heterogeneously use the means and indicators (levels of use, health, welfare, antimicrobial resistance, etc.) that allow them to adapt their practices in terms of treatment choices and farm management.”

#### 3.3.1 Problems related to consumption

**Fig 3:**
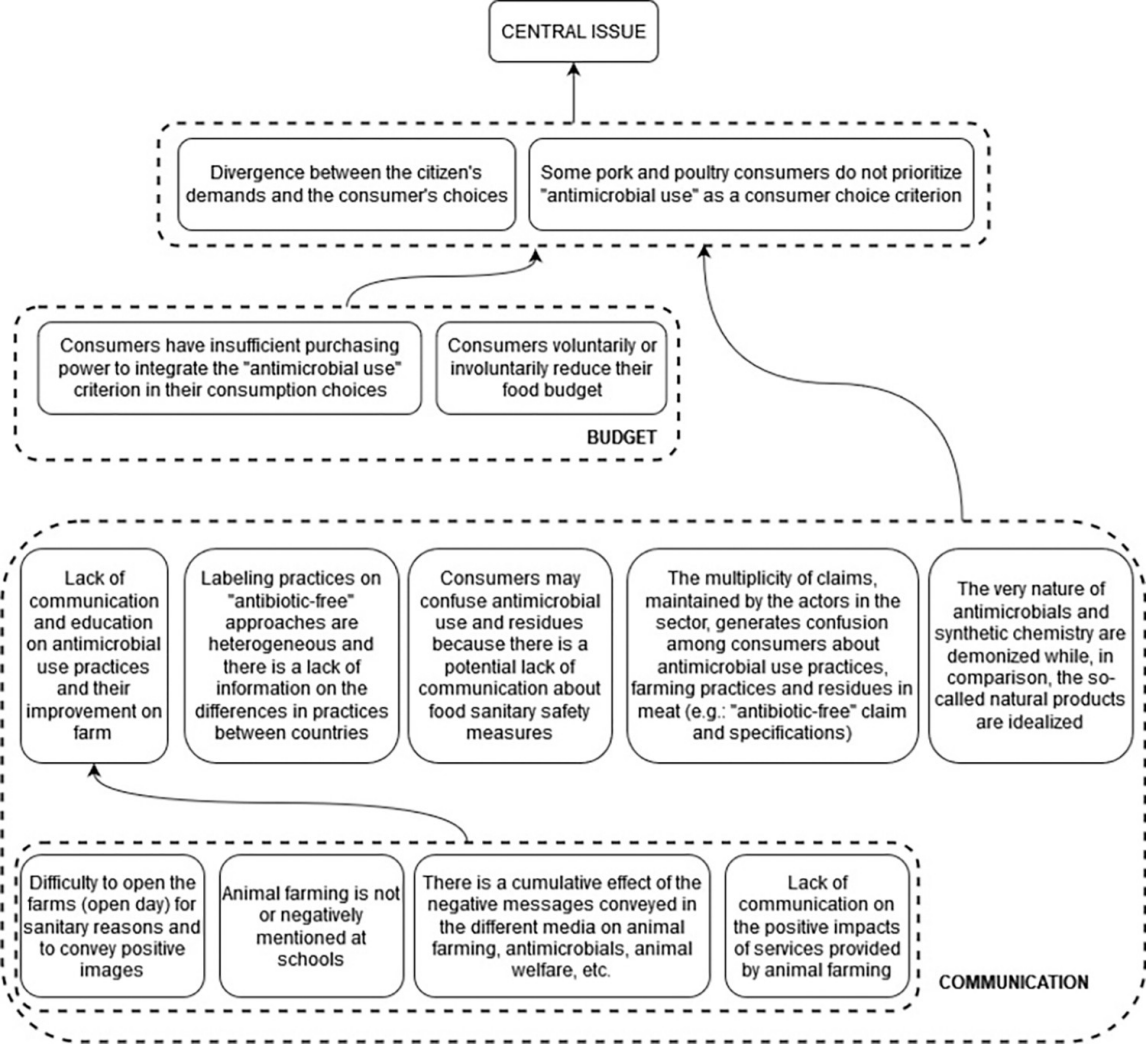
Root of the problem tree related to consumption and contributing to the antimicrobial use issue in pig and poultry sectors in France.

The group of participants identified thirteen problems related to consumption aspects and the fact that some consumers do not prioritize AMU reduction as a choice criterion despite the citizens’ interest in products considered healthier and better for the environment. According to the participants, consumers do not prioritize AMU as a choice criterion because of underlying causes related to budget or communication. The participants explained the fact that consumers do not integrate the AMU choice criterion because they voluntarily or involuntarily reduce their food budget due to insufficient purchasing power. The nine underlying causes related to the consumer communication reflect the fact that the lack of communication and education, the negative messages conveyed on AMU, AMR, farm management and food sanitary safety, as well as the multiplicity of food labels and claims generates confusion among consumers who cannot therefore choose with full knowledge (Fig 3).

#### 3.3.2 Problems related to competitiveness

**Fig 4:**
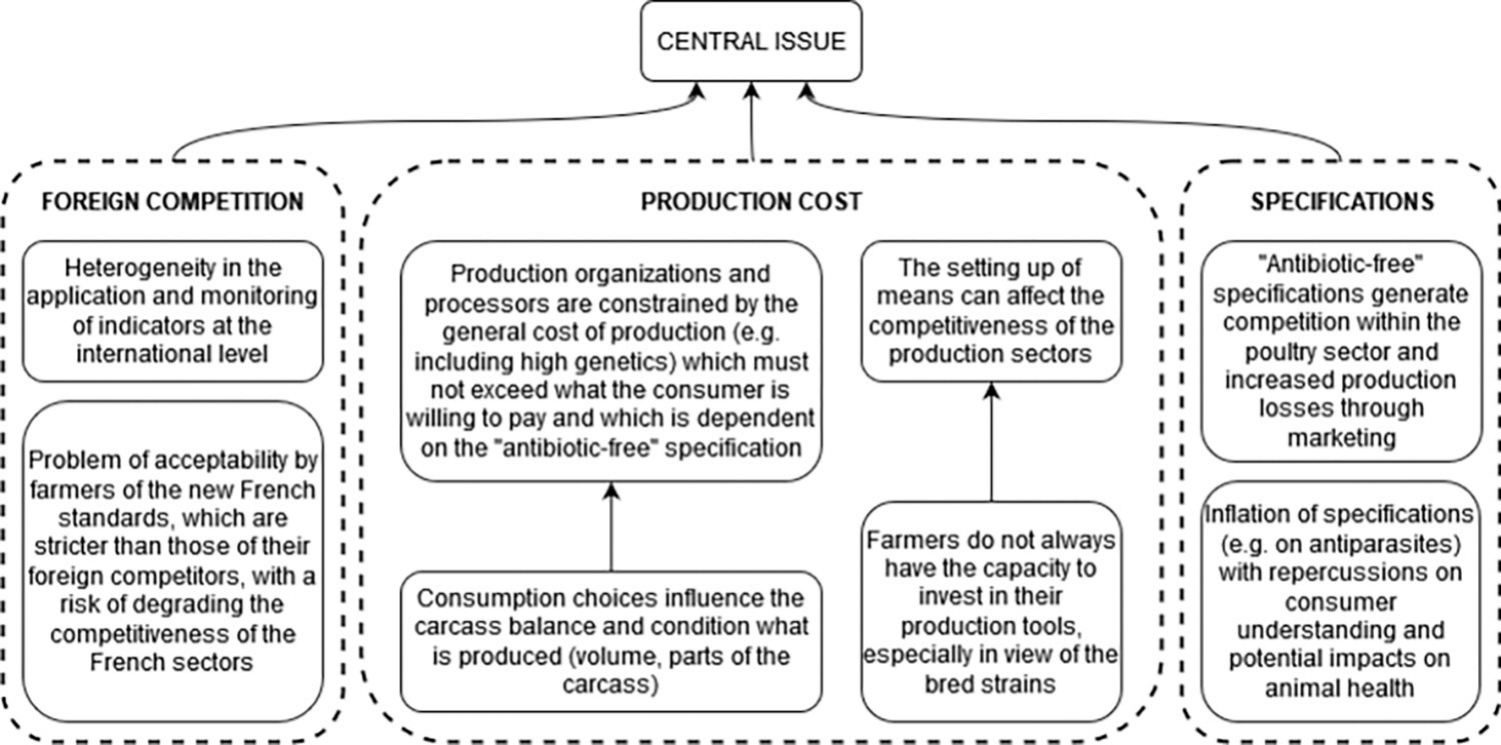
Root of the problem tree related to competitiveness and contributing to the antimicrobial use issue in pig and poultry sectors in France.

The participants mentioned eight problems related to competitiveness aspects and the fact that the French pig and poultry sectors are in competition with other countries that apply less strict production standards and controls, making it more difficult for farmers to accept French standards. To remain competitive, production organizations and processors must satisfy consumer choices in terms of product and price that constrain them to reduce the production cost and do not allow better AMU. The marketing initiatives foster the inflation of specifications, such as the "antibiotic-free" labels that generate production losses and competition, particularly within the poultry sector where there is a devaluation risk of the whole batch if an AM treatment is needed. These specifications regulate farming and treatment practices that can generate negative impacts on animal health which are often unknown by consumers. In addition, the implementation of means to reduce AMU increases production costs and requires an investment capacity that farmers do not always have (Fig 4).

#### 3.3.3 Problems related to indicators and monitoring

**Fig 5:**
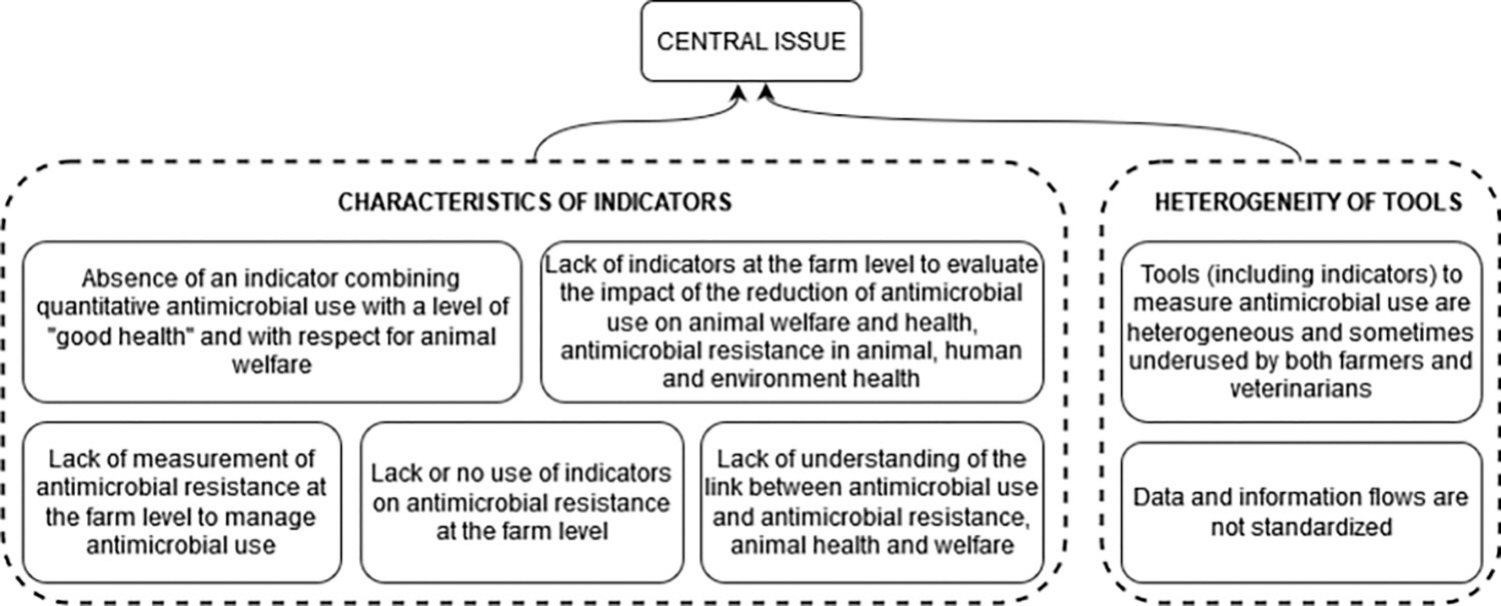
Root of the problem tree related to indicators and monitoring and contributing to the antimicrobial use issue in pig and poultry sectors in France.

The participants mentioned seven problems related to indicators and monitoring and the fact that the characteristics of indicators or their use are currently not optimal despite their multitude. There are no indicators to evaluate the impact of AMU at the farm level on animal health and welfare and on AMR in animal health, public health and the environment, which are the targeted impacts. There is also a lack of an indicator that would combine AMU with animal health and welfare and AMR at the farm level and that would allow actors to adapt practices more precisely. The group also mentioned that the indicators and the data exchange flows are heterogeneous and not standardized between production organizations, making it difficult to monitor them and therefore to adapt practices (Fig 5).

#### 3.3.4 Problems that influence changes in veterinary and farming practices

**Fig 6:**
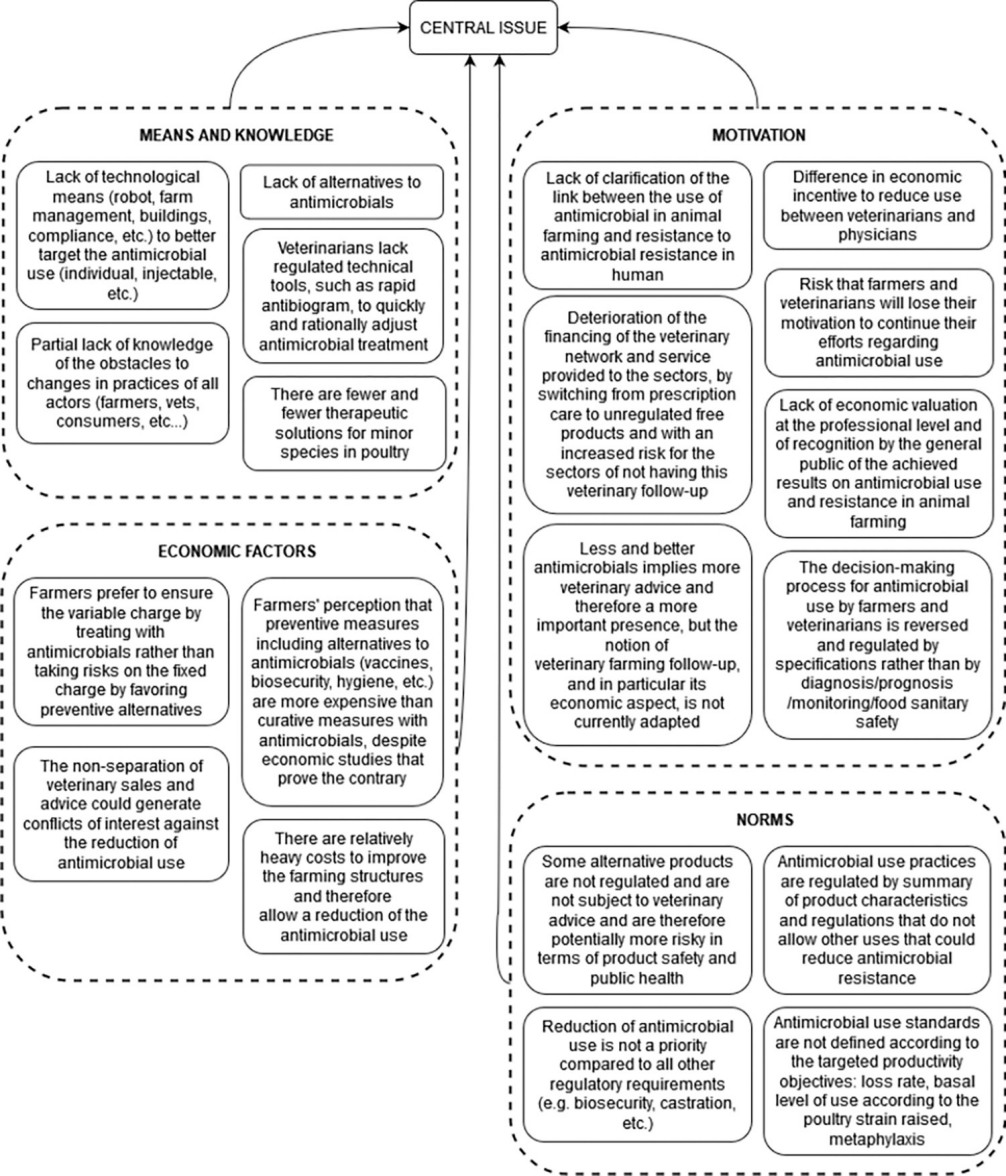
Root of the problem tree related to causes that influence changes in veterinary and farming practices and contributing to the antimicrobial use issue in pig and poultry sectors in France.

The participants mentioned twenty problems, divided into four subgroups, that influence changes in veterinary and farming practices (Fig 6). The group identified five problems due to a lack of means or knowledge, including the lack of technical and technological means to adapt AM treatment, the lack of alternatives to AMs, and the partial lack of knowledge of other levers of change.

They identified four economic factors such as the fact that farmers prefer to treat with AMs when necessary rather than invest in preventive alternatives which are perceived as more expensive despite economic studies showing the opposite. They mentioned once more the fact that the cost for improving farming infrastructures (necessary for better AMU) is high. The participants wanted to list, although not out of conviction but for the sake of exhaustivity, the potential conflict of interest due to the non-separation of veterinary sales and advice.

The participants mentioned seven problems related to the risk that farmers and veterinarians lose their motivation to continue their efforts regarding AMU. The deterioration of the financing of the veterinary network and service, including veterinary farm follow-up and advice, could demotivate the veterinarians whereas their role is essential to improve and reduce AMU. The farmers and veterinarians could also be demotivated because they have less decision power as the production organizations’ specifications drive AMU rather than their expertise. The participants also mentioned the lack of economic valuation and incentives and the lack of recognition by the general public of the achieved results on AMU and AMR in animal farming. As mentioned in the group of problems related to indicators, the lack of clarification of the link between AMU in animal farming and AMR in human health, which is the targeted impact, could demotivate animal health and production actors to maintain their efforts.

The participants identified four problems related to norms. The current AMU standards and norms are not compatible with the targeted productivity objectives and does not allow other AMUs that could reduce AMR. Reduction of AMU is not a priority compared to all other regulatory requirements (e.g. biosecurity, castration, etc.) the farmers have to achieve. Some alternative products are not regulated and are not subject to veterinary advice and could potentially generate negative impacts in terms of product safety for public health and economic shortfalls for the veterinary activity.

### 3.4 Ecosystem and scope of the intervention

In the third PW, the participants selected and included twenty-four problems in the scope of the intervention. The participants felt legitimate and in capacity to address five problems related to communication, two problems related to competitiveness, the entire group of problems related to indicators and monitoring and ten problems that influence changes in veterinary and farming practices. In the fourth PW, we proposed to group and reformulate interrelated and similar problems to avoid duplication of strategy identification. For example, similar economic problems were mentioned in the groups of problems related to competitiveness and barriers to change. Several problems related to communication mentioned different aspects to include in messages to the general public and could be addressed together. Likewise, several problems related to indicators mentioned different criteria to include in their definition and could be addressed together. The participants revised some of the twelve proposals and validated fifteen final formulations (Table 1). The group prioritized the resolution of the problems related to indicators and monitoring because it is a necessary prerequisite for the resolution of other problems related to specifications, communication on practices and impacts, veterinary and farming practices. Among the intervention ecosystem, the participants identified synergies with other projects under construction working on this issue such as the third Ecoantibio plan and the Calypso initiative. The collective decided to work on economic and communication related issues in a second phase with the potential help of other actors and experts.

**Table 1:** List of the problems included in the scope of the intervention in order of priority.

**Problems related to indicators and monitoring**
*“Lack or misuse of standardized indicators (for means and results) that*, *when properly combined*, *allow for the adaptation of AMU at the farm level with respect to the objectives in terms of animal health and welfare*, *AMR in livestock production*, *competitiveness*, *impact on public health and the environment*.*”*
*“The data to calculate the indicators*, *the means of collecting these data and the data exchange flows are heterogeneous and sometimes under- or misused by livestock farmers*, *veterinarians and others*.*”*
*« Lack of AMU observance*.* »*
**Problems related to economic**
*“Farmers prefer to ensure the variable charge by treating with AMs rather than taking risks on the fixed charge by favoring preventive alternatives because they think that preventive measures including alternatives to antimicrobials (vaccines*, *biosecurity*, *hygiene*, *etc*.*) are more expensive than curative measures with antimicrobials*, *despite economic studies that prove the contrary*.*”*
*“There are relatively heavy costs to improve the farming infrastructure and thus allow a reduction of AMU and farmers lack investment capacity to improve their production tools and infrastructure*.*”*
*“Less and better antimicrobials imply more veterinary advice and follow-up and therefore a more important presence*, *but the notion of veterinary farm follow-up*, *and in particular the economic aspect*, *is not currently adapted and there is a deterioration of the financing of the veterinary network and service provided to the sectors*, *by switching from prescription care to unregulated free products*.*”*
*““Antibiotic-free” specifications*, *supported by marketing*, *generate competition within the poultry sector and increased production losses and reverse the decision-making process for the AMU by farmers and veterinarians is reversed which is regulated by specifications rather than by diagnosis/prognosis/monitoring/food safety*.*”*
**Problems related to communication**
*“The multiplicity of claims (“antibiotic-free” claims and specifications) maintained by the actors in the sector*, *generates confusion among consumers about farming practices*, *animal health and welfare*, *the very nature of AMs and synthetic chemistry*, *the AMU practices*, *the food sanitary safety and residues in meat*.*”*
*“The cumulative effect of the negative messages conveyed in the different media generates confusion among consumers about farming practices*, *animal health and welfare*, *the very nature of AMs and synthetic chemistry*, *the AMU practices*, *the food sanitary safety and residues in meat*.*”*
*“Difficulty to open the farms (open day) because of previous decisions taken for sanitary reasons and to convey positive images*.*”*
*“Lack of education on AMs versus natural products and risks of residues in meat*.*”*
**Problems related to norms**
*“AMU standards are not defined according to the targeted productivity objectives*: *loss rate*, *basal level of use according to the poultry strain raised*, *metaphylaxis*.*”*
**Problems related to motivation to change of practices**
*“Lack of clarification of the link between AMU in animal husbandry and AMR in humans*.*”*
**Problems related to the lack of means**
*“Lack of technological means (robot*, *farm management*, *buildings*, *compliance*, *etc*.*) to better target the AMU (individual*, *injectable*, *etc*.*)*.*”*
*“Veterinarians lack regulated technical tools*, *such as rapid antibiogram*, *to quickly and rationally adjust AM treatment*.*”*

AMU, antimicrobial use; AMR, antimicrobial resistance; AMs, antimicrobials.

### 3.5 Typology of actors

During the third PW, we asked participants to identify the actors who are protagonist and/or impacted by the problems included in the scope of the intervention in order to resolve problems in regard with their strategies. The participants identified and characterized seventeen groups of actors directly or indirectly associated with the problems selected in the scope of the intervention, whose potential changes in practices, behavior and interactions could influence the resolution of the above-mentioned problems. This identification included actors involved in AMU (veterinarians, farmers, production organizations, pharmaceutical industries, etc.) and actors involved in the use of animal products (slaughterhouses, distribution, restaurants, consumers, etc.) but also governmental institutions, research and academic actors, non-governmental organizations (NGOs) and media (Table 2). According to the participants, the Ministry of Agriculture and Food and the Ministry of Health are major and influential actors as promoters of initiatives that have to be involved in the intervention. Animal health and production actors are also major in the innovation process and in achieving the targeted changes. Researchers have a major role in the production of the necessary scientific data and in the implementation of the intervention within the LL. For the resolution of problems related to alternatives, the pharmaceutical industry has to be involved, and for problems related to communication, the press and media as well. Some actors may react and influence the innovation process differently, even within the same category, being either in opposition or contribution. Similarly, some actors may be positively or negatively impacted by the resolution of the selected problems. The participants had to consider these differences in the construction of adapted strategies, to neutralize negative/antagonist forces and/or to guarantee the success of the intervention.

**Table 2:** List and typology of actors associated with the scope of the intervention.

	Actors	Type	Homogeneity	Impact	Contribution
**Governmental institutions**	Ministry of Agriculture and Food DGAL Ministry of Health	Major Influential Impacted	Homogenous	+	C
	Ministry of Ecological Transition and Solidarity	Impacted	Homogenous	+	N
	ANSES (risk evaluation) ANMV (regulation)	Major Influential Impacted	Homogenous	+	C
**Veterinarians**	Veterinary analysis laboratory	Major Impacted	Homogenous	+	C
	SNGTV Unions of Veterinarians Veterinarians	Major Influential Impacted	Heterogenous	+/-	C
**Animal production actors**	Inter-branch organizations Unions of the different professions	Major Influential Impacted	Heterogenous	+	C
	Farmers, including independent and short circuit	Major Influential Impacted	Heterogenous	+/-	C/O
	Production organizations Animal production operators Cooperatives	Major Influential Impacted	Heterogenous	+/-	C/O
**Buyers consumers**	Buyers of the processing and distribution Slaughterhouses Out-of-home catering	Major Influential Impacted	Heterogenous	+/-	C/O
	Consumers’ associations	Influential Impacted	?	+	C/N
**Research and academics**	National Veterinay Schools (ENV) Actors in education and training	Influential Impacted	Heterogenous	+	C
	Survey institutes Technical and scientific research (ANSES, INRAE, ITA, ENV)	Major Influential	Heterogenous	?	C
**Pharmaceutics**	Laboratories manufacturing antimicrobials, SIMV	Major Impacted	Heterogenous	+/-	C/O
	Sellers of alternatives	Impacted	Heterogenous	+/-	C/O
**Media**	Professional and general press Ambassadors Social networks press and media	Major Influential	Heterogenous	+	C/O
**NGOs**	Welfarist NGOs	Influential	Heterogenous	?	C/O
**Others**	Equipment manufacturers Installers	Influential Impacted	?	+	C

+, positively impacted by the intervention; -, negatively impacted by the intervention; C, in contribution to the intervention; N, neutral position regarding the intervention; O, in opposition to the intervention; DGAL, General Directorate of Food; ANSES, National Agency for Food, Environmental and Occupational Health & Safety; ANMV, National Agency for Veterinary Medicines; SNGTV, National Society of Veterinary Technical Groups; INRAE, National Research Institute for Agriculture, Food and Environment; ITA, Agricultural Technical Institutes; SIMV, Union of the Veterinary Medicine Industry (syndicate of antimicrobials manufacterers); NGOs, Non-Governmental Organizations.

### 3.6 Outcomes mapping

During the fourth PW, the participants drew the outcomes mapping to solve the following priority problem: “*Lack or misuse of standardized indicators (for means and results) that*, *when properly combined*, *allow for the adaptation of AMU at the farm level with respect to the objectives in terms of animal health and welfare*, *AMR in livestock production*, *competitiveness*, *impact on public health and the environment*.”. The sections 3.6.1–3.6.6 described the different theories of change identified by the participants.

#### 3.6.1 A One Health approach to conduct an AMR risk assessment

**Fig 7:**
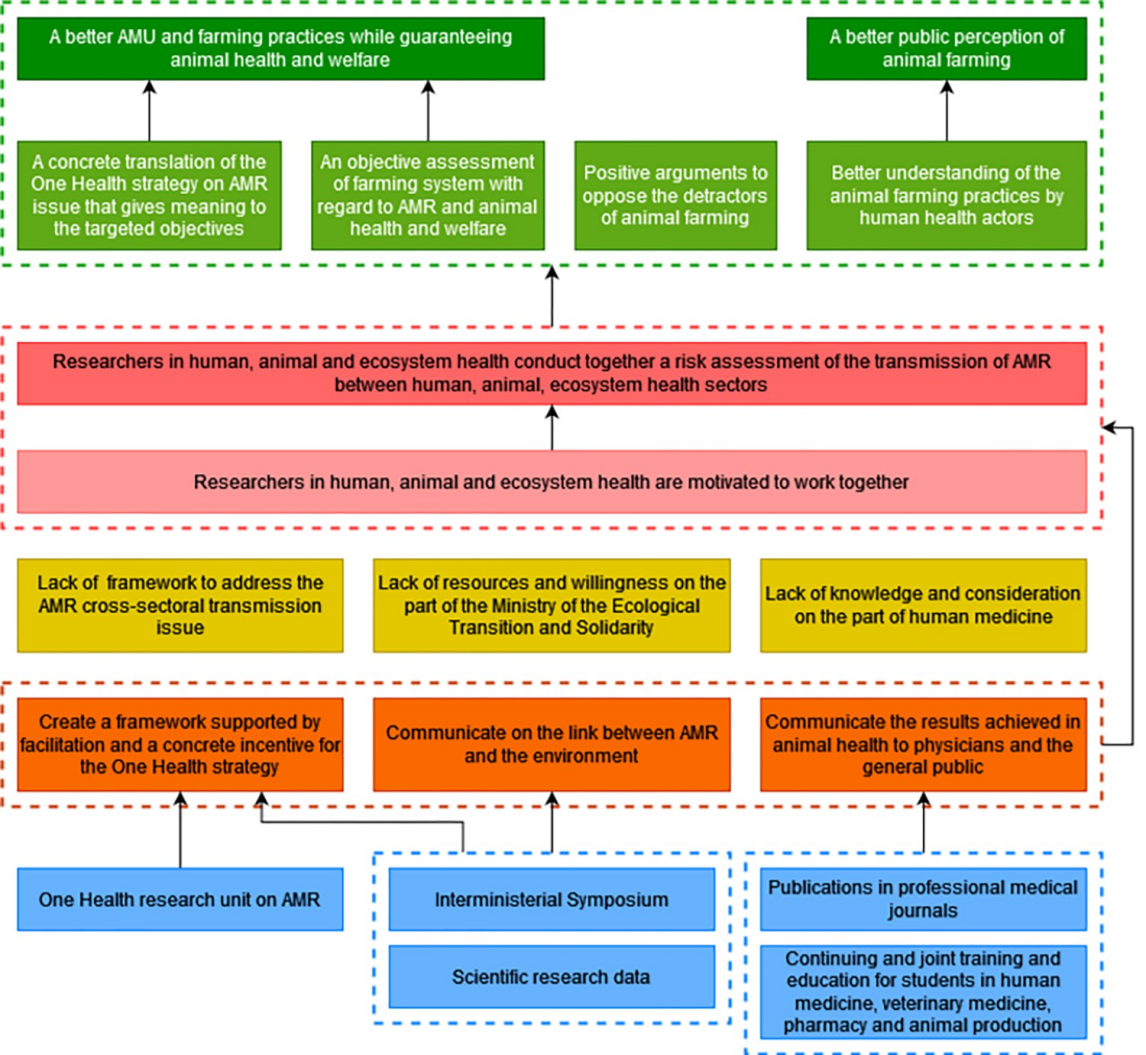
First branch of the outcome map: A One Health approach to conduct an antimicrobial resistance risk analysis. Dark green boxes, long-term impacts; Pale green boxes, medium-term impacts; Dark pink boxes, final changes in terms of interactions, behaviors and practices; Pale pink boxes, intermediate changes in terms of knowledge, capacities and motivations; Yellow boxes, current obstacles in terms of contextual factors or actors to generate the desired changes; Orange boxes, strategies to overcome the obstacles; Blue boxes, intervention outputs and activities to implement these strategies; AMR, antimicrobial resistance; AMU, antimicrobial use.

Participants mentioned that the proper use and the development of indicators must be based on scientific consensus regarding the link between AMR in animal, human and ecosystem health. This requires that researchers from these three health sectors be motivated to conduct together a risk assessment of the AMR cross-sectoral transmission. The lack of framework to address this issue may be a barrier to these changes in motivation and interaction. To overcome this obstacle, a facilitation strategy supported by a concrete political incentive may create the favorable framework for this collective and integrated approach. To encourage policy-makers to foster a One Health strategy, it is necessary to produce research data on the AMR cross-sectoral transmission evidence. The One Health interministerial symposium on AMR, which has been impacted by the Covid-19 pandemic, and the creation of a One Health research unit working on AMR could provide an enabling environment for desired changes. According to the participants, and despite the intersectoral meetings, the lack of knowledge and consideration among human health actors of the key role of animal health actors in the fight against AMR could demotivate them to work together. To overcome this obstacle, communication strategies need to be implemented to share the results achieved in animal health on AMU, to physicians and ideally to the general public. This could be achieved through publications in professional journals for physicians or through continuing and joint training and education between veterinary and animal production students and medical and pharmacy students. The ecosystem health sector is still poorly involved in the fight against AMR and there is still a lack of resources and a lack of willingness on the part of the Ministry of Ecological Transition and Solidarity (METS) to collaborate on this issue. Providing scientific evidence of the link between AMR and its impact on the environment could motivate the METS to get more involved in the One Health strategy. The participants mentioned that the targeted changes would contribute to improve AMU and farming practices while guaranteeing animal health and welfare thanks to the concrete translation of the One Health strategy on the AMR issue that would give meaning to the targeted objectives in animal health and therefore would motivate the actors of the sector to improve their practices and thanks to an objective assessment of each farming system regarding AMR and animal health and welfare. The risk analysis would give positive arguments to oppose the detractors of animal farming and would therefore contribute to defend the pig and poultry sectors. By working with the animal health sector, the human health actors would have a better understanding of what is done in animal health and this would contribute to improve the public perception of animal farming (Fig 7).

#### 3.6.2 Multi-actors working groups to identify new indicators

**Fig 8:**
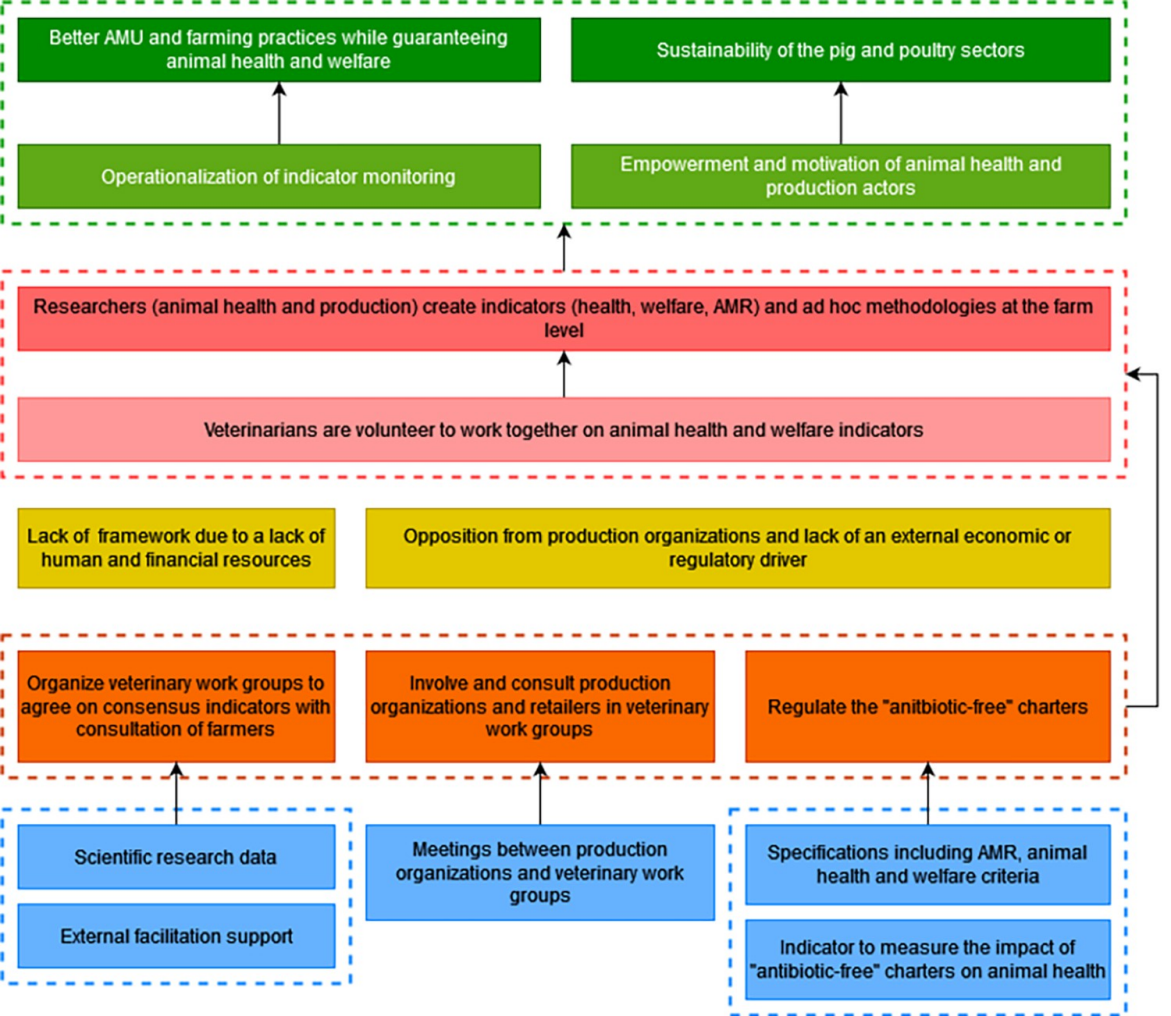
Second branch of the outcome map: Multi-actors working groups to identify new indicators. Dark green boxes, long-term impacts; Pale green boxes, medium-term impacts; Dark pink boxes, final changes in terms of interactions, behaviors and practices; Pale pink boxes, intermediate changes in terms of knowledge, capacities and motivations; Yellow boxes, current obstacles in terms of contextual factors or actors to generate the desired changes; Orange boxes, strategies to overcome the obstacles; Blue boxes, intervention outputs and activities to implement these strategies; AMR, antimicrobial resistance; AMU, antimicrobial use.

To contribute to the resolution of the problem, veterinarians must volunteer to work together and in collaboration with animal health and production researchers to create new indicators that are appropriate and informative of AMR and animal health and welfare at the farm level. These indicators are currently not explored by veterinary associations partly due to the fact that there is a lack of framework for collective reflection on this issue and a lack of financial and human resources to implement it. To overcome this problem, working groups including veterinarians should be organized to find a consensus definition of animal health and welfare indicators. The participants mentioned that the adaptability of indicators depends on the veterinarians’ field and animal health expertise and that their acceptability and practicability must be assessed by consulting farmers. It is necessary to provide an external facilitation support that guarantees veterinary ownership in these working groups, and research data on indicators to implement this strategy. Another obstacle to the creation of new indicators is the fact that there is no downstream demand from the production organizations and retailers who may perceive this initiative as a hindrance to the development of the sectors and may therefore oppose the intervention. To minimize the opposition of production organizations and retailers on the creation of new indicators, they must be involved and consulted on what will be produced by the veterinary working groups. This obstacle is also linked to the fact that production organizations are under economic pressure that induces them to do the minimum required in absence of economic or regulatory incentives to produce differently with regard to AMU. A regulation of the "antibiotic-free" labels was identified as a strategy to overcome this obstacle. To influence policy-makers to implement this regulation, an indicator of neglected care should be defined to provide evidence that the current "antibiotic-free" labels may have negative impacts on animal health and welfare. Health, welfare, and AMR oriented charters that reciprocally engage the farmers and producers’ responsibility in terms of obligation of means and the veterinarians’ responsibility in terms of performance obligation were also identified as a needed output. Achieving these desired changes would contribute to the operationalization of indicator monitoring that would lead to a better AMU while guaranteeing animal health and welfare. They would also contribute to the empowerment and motivation of animal health and production actors to continue their activity and therefore contribute to the sustainability of the pig and poultry sectors (Fig 8).

#### 3.6.3 A multi-actors agreement on the use of combined indicator

**Fig 9:**
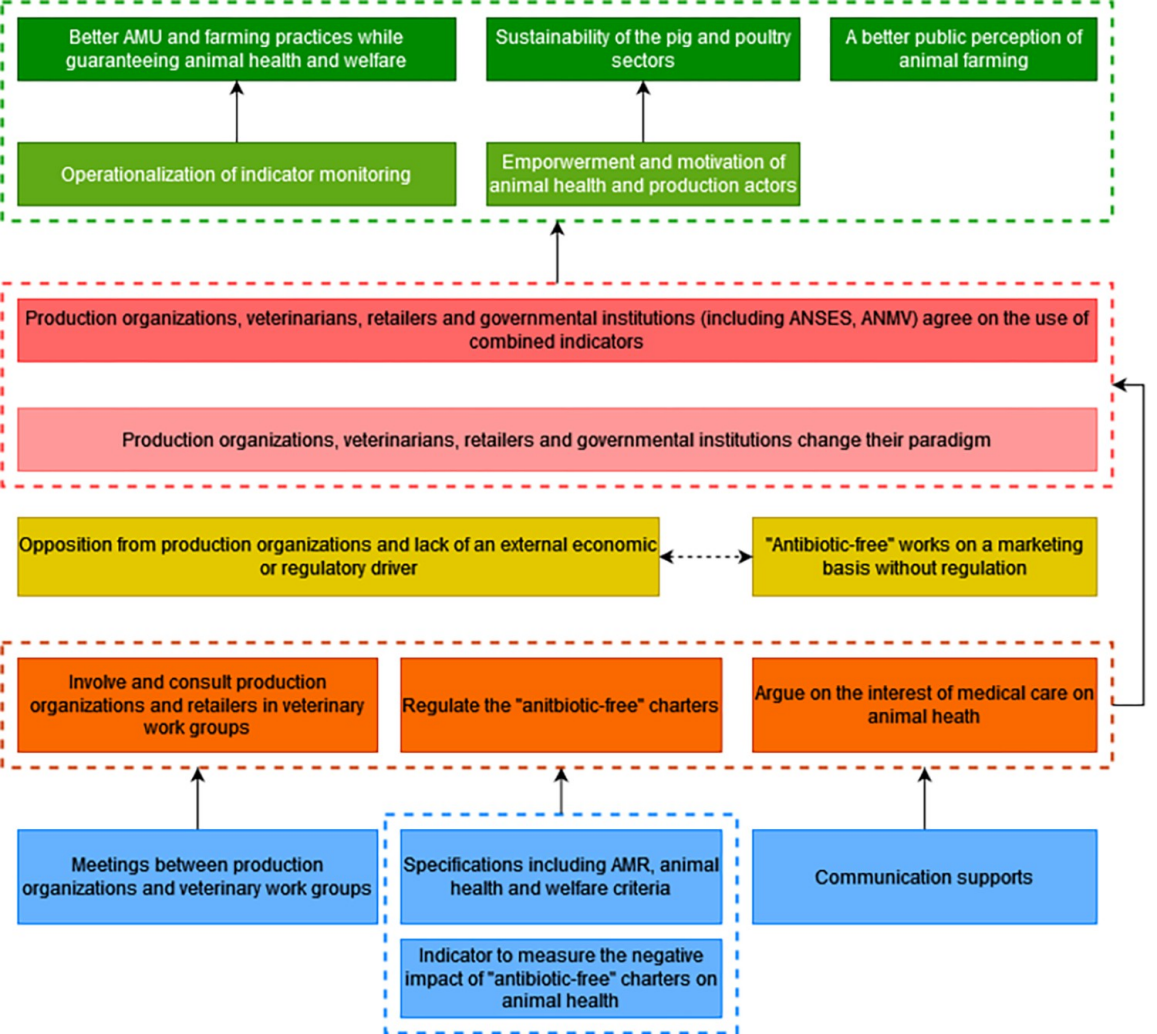
Third branch of the outcome map: A multi-actors agreement on the use of combined indicator. Dark green boxes, long-term impacts; Pale green boxes, medium-term impacts; Dark pink boxes, final changes in terms of interactions, behaviors and practices; Pale pink boxes, intermediate changes in terms of knowledge, capacities and motivations; Yellow boxes, current obstacles in terms of contextual factors or actors to generate the desired changes; Orange boxes, strategies to overcome the obstacles; Blue boxes, intervention outputs and activities to implement these strategies; AMR, antimicrobial resistance; AMU, antimicrobial use.

To contribute to the resolution of the problem, production organizations, including farmers, veterinarians, retailers, governmental institutions, including the National Agency for Food, Environmental and Occupational Health & Safety (ANSES) and the National Agency for Veterinary Medicines (ANMV), must agree on the best way to combine newly created and/or existing indicators. There are currently various AMU indicators but they are different between production organizations. These data could be combined and leveraged at the national scale to be statistically meaningful. Actors need to change their paradigm to use indicators differently. "Antibiotic-free" claims are based on effective but unregulated marketing strategies. Therefore, in the absence of economic or regulatory incentives, production organizations have no reason to change this paradigm and may oppose these changes. To minimize the opposition of production organizations and retailers to the combination of new indicators, the regulatory strategies identified in section 3.6.2 would contribute to overcome this obstacle. The participants identified a communication strategy to argue for the benefits of medical care, including AM treatment, on animal health. This requires to produce communication materials with a message that addresses the issues identified in the communication-related problems (section 3.3.1). Achieving the desired changes would contribute to the same impacts identified in the section 3.6.2. In addition, the paradigm shift and the communication tools produced would contribute to improve the public perception of animal farming (Fig 9).

#### 3.6.4 Use of existing techno-economic indicators

**Fig 10:**
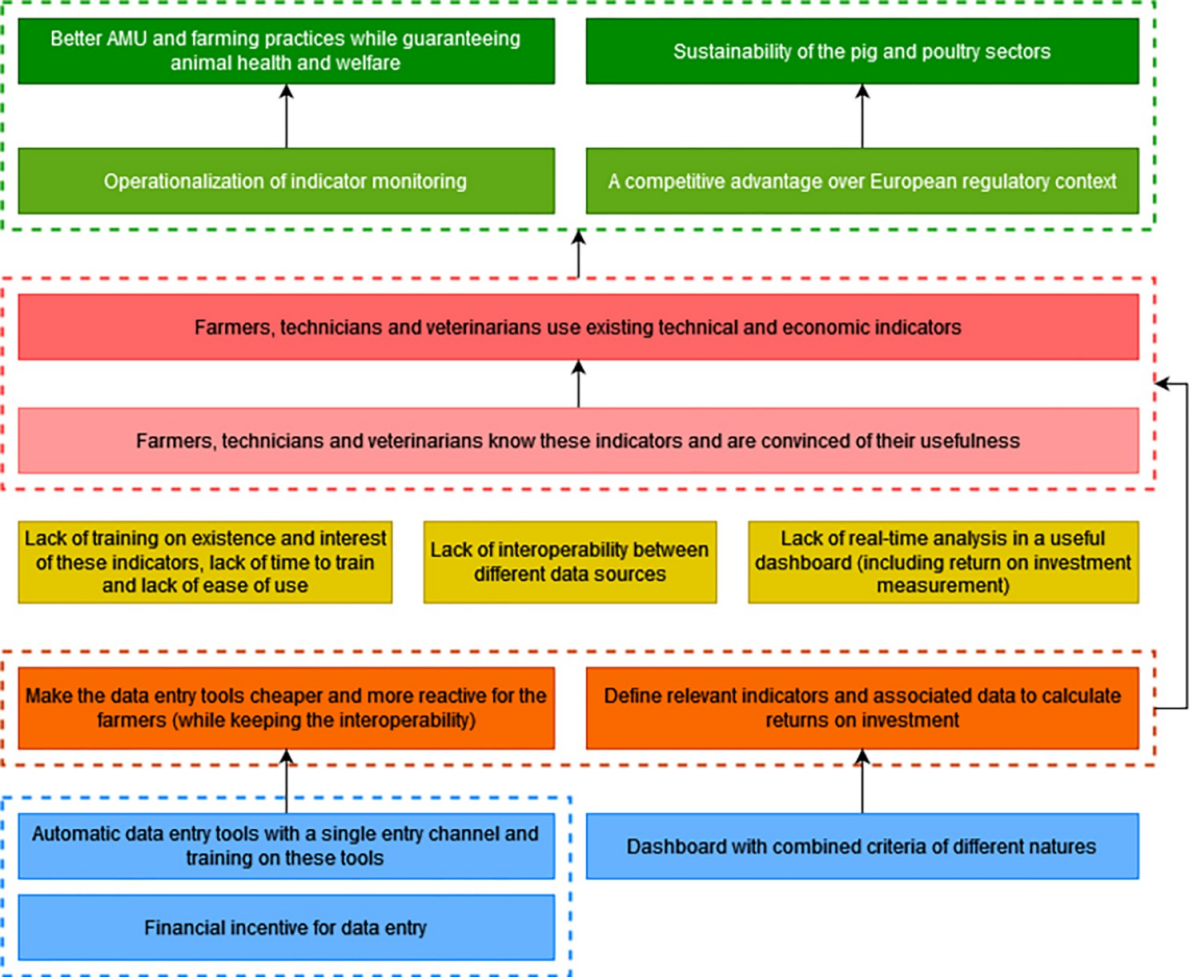
Fourth branch of the outcome map: Use of techno-economic indicators. Dark green boxes, long-term impacts; Pale green boxes, medium-term impacts; Dark pink boxes, final changes in terms of interactions, behaviors and practices; Pale pink boxes, intermediate changes in terms of knowledge, capacities and motivations; Yellow boxes, current obstacles in terms of contextual factors or actors to generate the desired changes; Orange boxes, strategies to overcome the obstacles; Blue boxes, intervention outputs and activities to implement these strategies; AMU, antimicrobial use.

To contribute to the resolution of the problem, farmers, technicians and veterinarians must use technical and economic indicators that already exist in most production organizations but are still underused. To make this change, they must be aware of their existence and convinced of their usefulness. Some of these actors currently lack training on the existence of such indicators or on their interest in using them. The lack of ease of use of these indicators can also be a barrier to their use, particularly in terms of digital interface and computer equipment for their entry. Some regions lack technical advice and training on data collection in the pig sector. In poultry, there is still technical advice, but technicians are going to do more and more tasks to the detriment of the time devoted to advice and training. There is also a lack of valorization of these indicators and of their analysis in real time to quickly adapt practices. There is also a lack of a dashboard that includes and analyzes different indicators such as the relevant ones to calculate the return on investment that would provide evidence to farmers of the economic benefits of investing in alternatives to AMs. Participants mentioned surveys with farmers that have revealed the problem of a lack of interoperability between tools to manage animal health that can discourage farmers from entering the same data in different indicator calculation software [40]. To overcome these obstacles related to the lack of ease of use of indicators, it is necessary to transform the data entry tools into a cheaper and more reactive version for the farmers while avoiding competition between production organizations that can worsen the problem of interoperability. Practical and automatic data entry and calculation tools with a single-entry channel and training on their use must be provided. It is necessary to set up a financial incentive to motivate farmers for data entry. In order to overcome the lack of interoperability and data valorization, it is necessary to define the relevant indicators and data to be included in the dashboard and particularly for the calculation of the return on investment. Achieving these desired changes would contribute to the operationalization of the monitoring of indicators that would lead to better AMU while guaranteeing animal health and welfare. This would also contribute to providing a competitive advantage and an advance on future European regulations and ultimately to foster the sustainability of the sectors (Fig 10).

#### 3.6.5 Standardization of data, indicators and analysis

**Fig 11:**
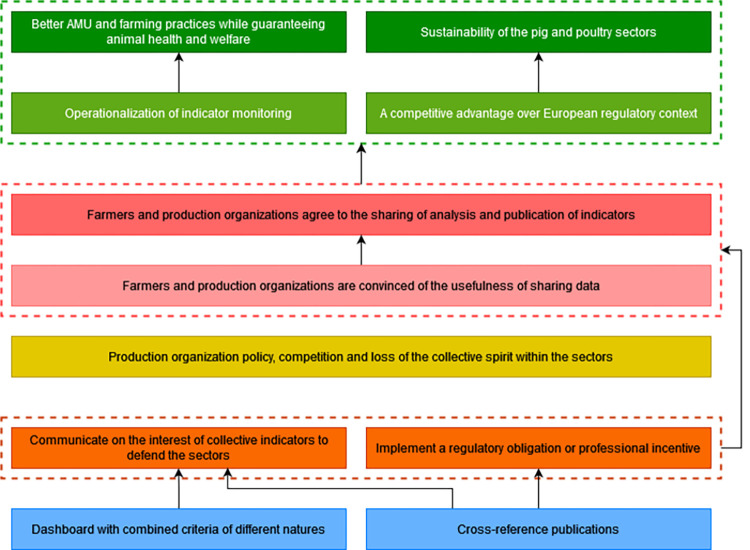
Fifth branch of the outcome map: Standardization of data, indicators and analysis. Dark green boxes, long-term impacts; Pale green boxes, medium-term impacts; Dark pink boxes, final changes in terms of interactions, behaviors and practices; Pale pink boxes, intermediate changes in terms of knowledge, capacities and motivations; Yellow boxes, current obstacles in terms of contextual factors or actors to generate the desired changes; Orange boxes, strategies to overcome the obstacles; Blue boxes, intervention outputs and activities to implement these strategies; AMU, antimicrobial use.

To contribute to the resolution of the problem, farmers and production organizations must be convinced of the advantage to share and standardize the analysis and publication of indicators at the national level. The current policy of production organizations and the competition within a sector can be an obstacle to change. There is also a loss of collective spirit in the sectors that need collective indicators to argue and defend themselves on the more global political issues that threaten the sectors. Communicating on the interest of these indicators and implementing a regulatory obligation or a professional incentive are levers to overcome the obstacle. The publication of cross-references is necessary to set up these strategies. It is also necessary to produce a dashboard that combines not only technical-economic indicators but indicators of different kinds. Achieving these desired changes would contribute to the same impacts described in section 3.7.4 (Fig 11).

#### 3.6.6 Commitment of administration and policy-makers

**Fig 12:**
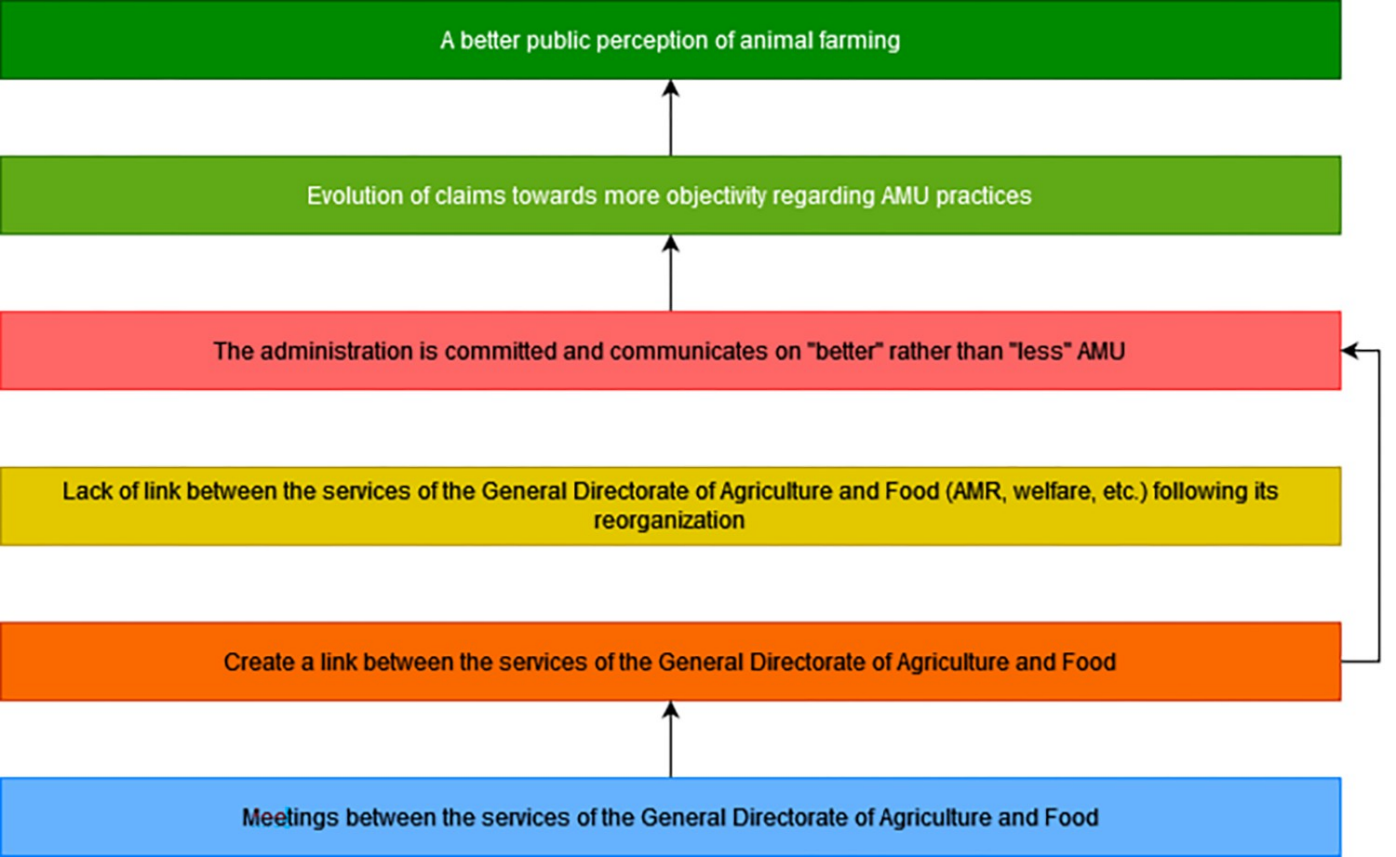
Sixth branch of the outcome map: Commitment of administration and policy-makers. Dark green boxes, long-term impacts; Pale green boxes, medium-term impacts; Dark pink boxes, final changes in terms of interactions, behaviors and practices; Pale pink boxes, intermediate changes in terms of knowledge, capacities and motivations; Yellow boxes, current obstacles in terms of contextual factors or actors to generate the desired changes; Orange boxes, strategies to overcome the obstacles; Blue boxes, intervention outputs and activities to implement these strategies; AMR, antimicrobial resistance; AMU, antimicrobial use.

To contribute to the resolution of the problem, the administration needs to engage and communicate on “better” rather than “less” AMU to positively influence the desirable changes previously identified. The Calypso project focuses mainly on quantitative data collection. There is a need for a strong political and administrative commitment, through the Ecoantibio 3 plan for example, in terms of financial incentives for research on the creation of indicators and in explaining to the general public and NGOs what good AMU is and which can therefore influence changes at the level of other actors. There is already willingness and communication on improved AMU but the potential lack of relationship between the services of the DGAL, resulting from its recent restructuring, may be an obstacle to this change. It is therefore necessary to create a link between these services working separately on health, welfare and AMR, by organizing joint meetings. Achieving the desired change would contribute to the evolution of claims towards a better public perception of animal farming and facilitate other changes mentioned above (Fig 12).

## 4. Discussion

Our study presents original results about implementing a participatory strategic planning approach to build an intervention that aims to improve AMU in livestock. The results of this study describe 1) an initial diagnosis of the current AMU situation in the pig and poultry sectors in France; 2) a common vision of the future to which participants would like to contribute through the intervention; 3) a systematic identification of the current problems opposed to this ideal situation of the vision of the future; 4) a defined scope of the intervention; 5) a typology of actors protagonist and/or impacted by those issues and 6) different branches of a global outcome map to solve a priority problem related to indicators and monitoring, that participants are going to use as a basis to build the action plan of their LL. Our study highlights the adaptability of the different steps of the ImpresS *ex ante* approach and provides insights on the plausible strategies that could be implemented in this particular context.

### 4.1 Towards a plausible and innovative impact pathway

We believe that the participatory strategic planning approach allowed the participants to be ambitious and innovative in the building of *ex ante* impact pathways towards a prudent AMU while identifying plausible and consistent objectives and strategies in regard with the particular context of the LL. The different steps of the ImpresS *ex ante* approach allowed for considering numerous contextual factors, anticipating different challenges to face and identifying the links between the needed outputs, strategies and outcomes to overcome these problems and to generate desired impacts. The approach is also actors-centered and allowed to consider this other level of complexity with regard to the intervention and to anticipate which actors to involve or partner with, and how actors could be impacted or opposed to the intervention. All these considerations allowed for an understanding of the complexity of the system in which the intervention is implemented and of the benefits and costs linked to the changes in practice. Therefore, it may have increased the plausibility of the outcome map towards better AMU that could be assessed in the evaluation phase of the LL [41].

The ImpresS *ex ante* approach allowed the participants to anticipate certain changes and to aim for a line of ambition that is beyond the zone of control and influence of the LL [35, 42]. The participants collectively formulated a vision of the future that is ambitious but necessary to address the issue of AMU in pig and poultry sectors in the most effective and holistic way. The objective of the intervention includes impacts on AMU practices, animal health and welfare, and sustainability of the pig and poultry sectors and of the veterinary network. To our knowledge this could be the first intervention that clearly aims to contribute to the achievement of such different impacts in a more systemic way. As noticed by participants in the initial diagnosis, previous initiatives aimed at reducing AMU. The first national Ecoantibio plan managed to reduce veterinary AMU by 37% between 2012 and 2016, 12% more than the targeted objective, and the second Ecoantibio plan aimed to continue these efforts between 2017 and 2021. However, quantitative AMU tends to stagnate and future interventions should target more qualitative AMU improvement goals, as formulated in the vision of the future [38]. Ethical considerations are also relevant to include to avoid negative impacts on animal health and welfare due to AMU reduction encouraged by labels. These marketing strategies are supported by the sectors and are influenced by uninformed consumption choices of consumers who may perceive “antibiotic-free” label as a promoter of healthier animals [43]. Pig and poultry sectors face an increasingly intense foreign competition that constrains cost of production and therefore necessary investment to improve AMU. The role of the veterinarian is central in improving AMU in livestock, but the maintenance of the veterinary network in rural areas is declining and the current economic model does not sufficiently value veterinary advisory services [44, 45]. These different socio-economic and ethical facets of the AMU issue are therefore connected and this is why it is relevant to consider them together and to include all this diversity of actors in the outcome targeted. To our knowledge, this is also the first time that an intervention that aims to improve AMU would target changes at the level of meat end users and not only at the level of AM users [43, 46].

The ImpresS *ex ante* approach allowed the participants to collectively identify underpinning causes that in their opinion impede the realization of the vision of the future, taking into account the complexity of those issues, but also to narrow down the scope of the intervention. The participants defined an ambitious scope of intervention that is coherent with the trajectory of change and the ecosystem of projects regarding AMU reduction in France. They felt legitimate and in capacity to address problems related to communication on AMR and AMU and farm practices, competitiveness stakes, barriers to change in veterinary and farming practices, and they prioritized the resolution on problems related to indicators and monitoring. The past interventions encouraged the emergence of various AMU indicators but that are not yet standardized and sometimes inappropriately used and the quantitative indicators, based on AM sales and on Animal Level of Exposure to Antimicrobials (ALEA), of the first Ecoantibio plan does not allow to assess the evolution of practices in veterinary medicine [39, 47]. Therefore, it is essential and innovative to have standardized and properly combined indicators to allow for the adaptation of AMU at the farm level with respect to the objectives in terms of animal health and welfare, and competitiveness, but also according to AMR in livestock production, public health and environment.

**Fig 13:**
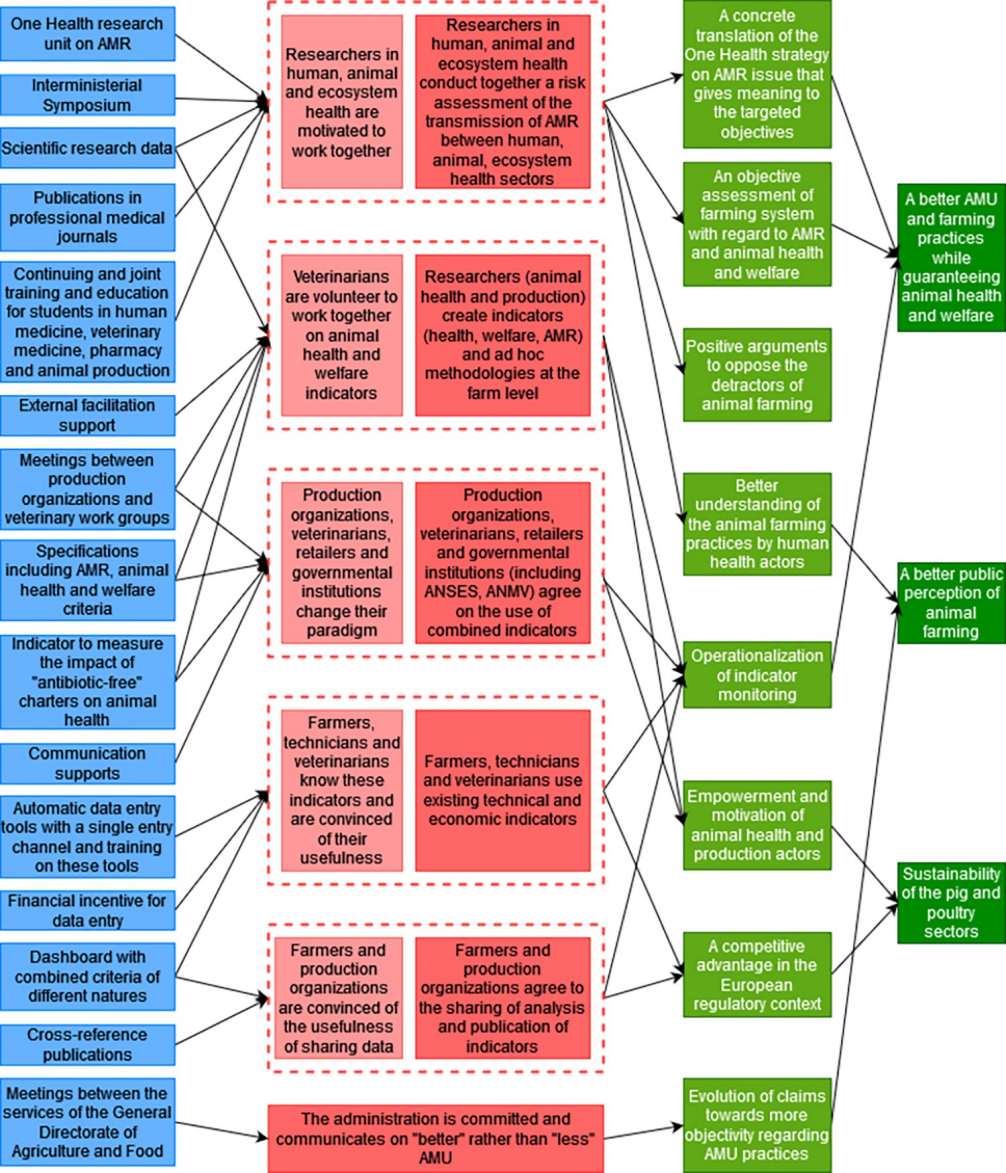
Impact pathway of an intervention towards prudent antimicrobial use in pig and poultry sectors in France. Dark green boxes, long-term impacts; Pale green boxes, medium-term impacts; Dark pink boxes, final changes in terms of interactions, behaviors and practices; Pale pink boxes, intermediate changes in terms of knowledge, capacities and motivations; Blue boxes, intervention outputs and activities to implement these strategies; AMR, antimicrobial resistance; AMU, antimicrobial use; ANSES, National Agency for Food, Environmental and Occupational Health & Safety; ANMV, National Agency for Veterinary Medicines.

The strategies identified by the participants to resolve the lack of such indicators and monitoring system are innovative and in line with the evolution of national and European recommendations and perspectives [47–49]. A One Health strategy is already promoted but needs to be strengthened to encourage researchers of the three health sectors to find a scientific consensus regarding the link between AMR in animal, human and ecosystem health, on which to base the development and use of appropriate indicators. Organizing working groups including veterinarians in consultation with farmers and production organizations is relevant to define the appropriate indicators and to organize this coherence in the framework of these highly integrated sectors [15, 16]. Participants also identified regulatory strategies to regulate "antibiotic-free" labels. In 2015, the Directorate General for Consumer Affairs (DGCCRF) initiated a group work to regulate these "antibiotic-free" labels by a decree. This decree aimed to harmonize and standardize the performance indicators, which differ between each sector and each company. This work has been suspended but remains relevant to anticipate a regulation by the public authorities and for the sake of consumer communication [50, 51]. Technological, communication and professional incentive strategies have also been identified. The involvement of the State seems essential to encourage the realization of all these strategies and changes as it was the case for the previous Ecoantibio plan. The iteration loops make it possible to verify the coherence of what is produced at each stage with respect to this vision of the future. In particular, we noticed that the different outputs would generate different interconnected changes that would partially contribute to the impacts formulated in the vision of the future (Fig 13).

### 4.2 From outcome maps to different outputs

The ImpresS *ex ante* approach made it possible to clarify the links between the impacts that the group wants to contribute to, the targeted changes that they think they can generate during the intervention, the obstacles to those changes, and the strategies and outputs needed to overcome those obstacles and generate the changes. This conceptual model makes it possible to clearly outline all these transition pathways. In our case study, some of the changes are under the direct influence of actors represented by the participants in the process. For these changes, the outcome map sections can be translated into action plans so that the participants can continue this collective work and test the strategies identified in the LL. For other parts of the outcome map, it would be required to involve other actors that have a more direct influence on the outcomes targeted such as production organizations, for example. In such cases, a compelling narrative of the intervention can be produced to support the engagement of policy-makers or other partners and raise interest in those issues. Finally, the identified outcomes can be the subject of a long-term change-oriented monitoring of the intervention to test the plausibility of those initial assumptions, and see what works, for whom, in what circumstances, and support the adaptive management of the intervention toward the vision of the future [29].

### 4.3 Benefits of the participatory approach

The main strength of this process lies in the use of the participatory strategic planning approach. Some interventions towards a prudent AMU include veterinarians or farmers in peer-learning, AMU stewardship and construction of herd and health management protocols, but to our knowledge, few integrate this level of participation from the design of the intervention [52, 53]. The ImpresS *ex ante* approach involved participation and collaboration of different relevant actors, considers different viewpoints, created a space of collective elucidation and learning in which participants were encouraged to mutually share their expertise and opinions on the different elements of the system all along the participatory process. This led to decrease the level of uncertainty regarding the outcome map towards better AMU in the pig and poultry sector in France. The ImpresS *ex ante* approach has been adapted in the framework of other LLs including different sectors in other partner countries of the ROADMAP project (https://www.roadmap-h2020.eu/). The participatory process resulted in a diversity of impact pathways with context-specificities and semi-regularities between the case studies [54, 55]. The participatory approach allowed to get actors involved in collective decision-making that increased their willingness to continue implementation efforts [56]. There were no apparent conflicts to manage during the process. All the participants listened to each other, but it could have been otherwise, and in this case the facilitation should have been adapted to manage the participants’ postures that could affect the quality of the construction process. We also think that participatory research projects, based on the LL method, and the exchanges inherent to the participatory approach are fruitful in lessons learned for research practices [57].

### 4.4 Limitations

The main limitation of our study, but common in participatory process, is the potential lack of representativeness of our results [58]. We did not involve other actors that may be concerned by the issue such as farmers, slaughterhouses, retailers, marketers, consumers, welfare organizations, pharmaceutical and animal feed companies, because of time constraints and to ensure the quality of the process without compromising participation and facilitation. We elected institutional actors who have a global expert vision of the functioning of the sectors and the interactions between actors. These actors had busy agendas and some of them could not participate to a few PWs. One participant attended only the first PW. To minimize absenteeism-related bias, we communicated reports of each PW and gave participants the opportunity to provide individual feedback and to discuss them with the research team or with the group of participants during each PW to validate previous results.

The application of such a participatory strategic planning approach to build an intervention is a long process that is not always compatible with the funding duration of research projects. The ImpresS *ex ante* approach is adaptative and flexible, and we made some adaptations at different levels. Because of time limitation and availability of participants, we had to organize several half day PWs instead of a full three days PWs. We chose to make proposals and to consult the participants for the formulation of the vision of the future, the central issue and the problems selected to be part of the scope of the intervention. We tried to deploy the method as completely and in a participatory fashion as possible and to iteratively discuss and validate the results produced in the previous steps. We thought that the time invested during the construction process is an investment made by the participants to facilitate the implementation of the intervention and to engage them in further activities of the LL. Because of the Covid-19 pandemic, we could not organize all meetings on-site, as initially planned. Instead, we used online collaborative platforms such as Zoom, Teams and Klaxoon, or hybrid workshops (partly distant and partly on site). Those adaptations could have impacted the participation and facilitation dynamic, resulting in potential lack of equality between participants, and the interpersonal relationships that are necessary to create a shared space for collaboration and trust between participants and with researchers [59, 60].

### 4.5 Recommendations and perspectives

At the end of this participatory process, and complementary to our discussion analyzing those results in the light of the existing literature, we recommend to assess the acceptability of the co-constructed strategies and the plausibility of the outcome map by interviewing other categories of actors that may be impacted or concerned by the intervention and by identifying contextual factors that may influence the intervention implementation. We plan additional PWs with participants to translate the outcome maps into a convincing narrative of the intervention logic and an action plan for partners and policy-makers. To be in line with the innovation trajectory, collaboration with public stakeholders who participated to this participatory process is necessary for generating the desirable impacts through public actions. We planned to experiment and assess the strategies through a LL research approach. Prior to implementing the intervention and based on impact indicators, we recommend to translate the outcome map, or part of it, into an outcome-oriented monitoring and evaluation system to tailor in real time the strategies and to gain insight on associated change processes [61, 62]. This participatory *ex ante* impact pathway building approach could be extended to other countries that aim to implement strategies towards a better AMU or to other type of interventions in agriculture.
